# Macrophage-Based Transcriptional Assays for the Comparative Assessment of the Anti-Inflammatory Paracrine Activity of Canine Adipose-Derived Mesenchymal Stromal Cells

**DOI:** 10.3390/biom16081190

**Published:** 2026-08-14

**Authors:** Andrea Exnerová, Sabina Seidlová, Věra Daňková, Vojtěch Pavlík, Kristina Nešporová

**Affiliations:** Cell Physiology Group, Contipro a.s., Dolní Dobrouč 401, 562 01 Dolní Dobrouč, Czech Republic

**Keywords:** mesenchymal stromal cells, macrophages, in vitro assays, assay standardisation, mesenchymal-stromal-cell-based therapy

## Abstract

Therapies based on mesenchymal stromal cells (MSCs) have high potential in the field of regenerative medicine due mainly to their immunomodulatory properties. However, their clinical translation is hampered by a lack of sufficiently standardised potency tests. Since macrophages constitute key mediators of the effects of MSCs, macrophage-based assays potentially provide a relevant in vitro tool for the evaluation of the activity of MSC products. This study involved the coculturing of canine adipose-derived mesenchymal stromal cells (ASCs) with macrophages derived from human THP-1 and U937 monocyte cell lines, murine RAW264.7 macrophages and primary human macrophages. The M2 polarisation was assessed following stimulation with IL-4/IL-13 in THP-1 and U937 macrophages. The mRNA expression of the pro- and anti-inflammatory markers was analysed using qPCR. The ASC transwell coculture altered the LPS-induced inflammatory mRNA expression in a strongly model- and marker-dependent manner. The U937-derived macrophages exhibited the most consistent suppression of the tested inflammatory transcripts and the RAW264.7 cells provided a practical readout for selected inflammatory markers, whereas the THP-1 macrophages evinced the suppression of *TNFA* but not *IL1B* or *PTGS2* under the selected stimulation conditions. IL-4/IL-13 induced moderate but statistically non-significant changes in *IL10* and *TGFB1* in the U937-derived macrophages but no reproducible response in the THP-1-derived macrophages. In a subsequent U937 coculture experiment, ASC-derived paracrine factors altered selected M2-associated transcripts at specific time points. The results thus provided support for macrophage-based transcriptional readouts as an early-stage tool for comparing responder macrophage models and detecting the selected anti-inflammatory paracrine effects of canine ASCs; the U937 cells were found to be particularly suitable for the study of inflammatory polarisation and the RAW264.7 cells for the purpose of standardised screening.

## 1. Introduction

MSCs are adult multipotent stromal cells that can be isolated from a wide range of tissues, including bone marrow, adipose tissue and dental pulp. The therapeutic potential of MSCs has been demonstrated in terms of a broad range of human and veterinary diseases, including osteoarthritis, chronic wounds, graft-versus-host disease (GvHD), Crohn’s disease and neurological disorders [1,2]. Extensive preclinical studies have further demonstrated that MSCs possess immunomodulatory and pro-regenerative properties [3]. ASCs consist of a subset of MSCs, and they exhibit regenerative properties that are broadly comparable to those of MSCs obtained from other sources.

Translating MSC therapy into clinical practice requires the application of standardised production and quality control approaches [4]. The International Society for Cell & Gene Therapy sets out minimum criteria for the characterisation of MSCs, including adherence to tissue culture plastic, the expression of CD105, CD73 and CD90, a lack of haematopoietic markers and trilineage differentiation potential [5]. The conventional clinical quality control approach primarily addresses identity, viability and sterility; however, donor-to-donor and batch-to-batch variability necessitate conducting additional potency tests [6,7,8]. Potency assays should be indication-specific and should, ideally, correlate with therapeutic efficacy.

The potency of MSCs can be assessed directly by applying gene, protein or surface marker analyses, or indirectly via functional assays that measure the impacts of MSCs on target cells [7,9,10]. Mixed lymphocyte reactions (MLRs) assess the interactions of MSCs with T cells and are relevant for diseases that are driven by adaptive immunity, such as GvHD. However, with respect to conditions such as osteoarthritis or chronic wounds, macrophages often constitute the key dysregulated immune cells, and MSCs are thought to primarily modulate macrophage phenotypes [11,12,13,14]. Consequently, the development of macrophage-based potency assays is steadily accelerating [15,16].

This study provides a comparative evaluation of human and murine macrophage responder models as exploratory transcriptional readouts of the paracrine activity of canine ASCs. The xenogeneic coculture was used to identify model- and marker-specific qPCR responses, and it was not the intention to create a species-matched potency-release assay. The primary relevance of the work lies in the development of an early-stage veterinary MSC assay and in reporting the general assay-design considerations.

## 2. Materials and Methods

### 2.1. Ethical Approval Statement

**Figure 1:**
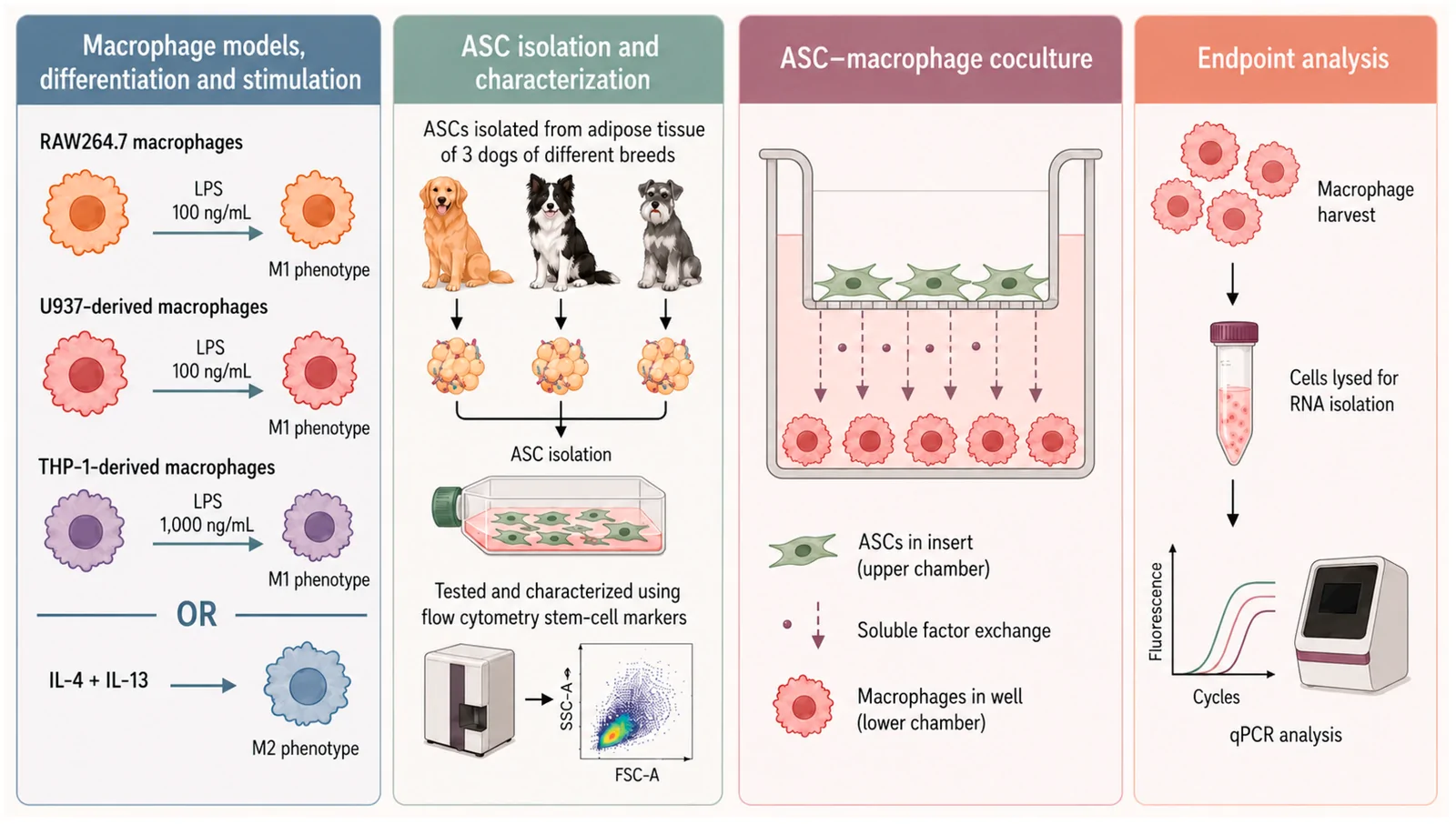
Schematic representation of the ASC-macrophage coculture experimental setup.

Adipose tissue (approximately 3 g) was obtained in the form of surplus material during routine, medically indicated veterinary procedures in client-owned dogs. No procedures were performed for research purposes. According to Czech legislation (Act No. 246/1992 Coll., on the Protection of Animals Against Cruelty), animals that are subjected to diagnostic or therapeutic interventions are not considered to be experimental animals; therefore, no approval was required from the Institutional Animal Care and Use Committee. All the veterinary procedures were performed by licensed veterinarians, and the owners provided their informed consent for the use of any surplus tissue for research purposes. All the experiments were performed in vitro using isolated cells. An overview of the cells used in the study and the experimental setup is provided in Figure 1.

### 2.2. Isolation and Culture of the Canine ASCs

The canine ASCs were isolated from the adipose tissue of three healthy client-owned female dogs that underwent routine ovariectomy. A summary of the donor characteristics is provided in Table A1. Fat tissue samples were obtained from the female dogs during the ovariectomies. A portion of the surplus visceral fat (approximately 3 g) was washed in 9.8 mL of physiological saline supplemented with 200 µL of penicillin–streptomycin (Diagnovum GmbH, Greifswald, Germany), minced and digested with 2 mg/mL of type I collagenase (Gibco, Grand Island, NY, USA) dissolved in αMEM (Capricorn Scientific GmbH, Ebsdorfergrund, Germany) containing 2% penicillin/streptomycin at 37 °C, 200 rpm for 60 min. The suspension was filtered through a 40 µm mesh, diluted with 10 mL of αMEM and centrifuged at 300 g for 5 min. The pellets were washed with physiological saline, resuspended in αMEM containing 10% FBS (Biosera, Cholet, France), 1% stable L-glutamine (Carl Roth GmbH + Co. KG, Karlsruhe, Germany) and 1% penicillin/streptomycin, and seeded in 150 cm^2^ flasks (total volume of 20 mL). The cells were cultured at 37 °C in 5% CO_2_, the medium was changed twice per week and the cells were passaged at 70–80% confluence. The ASCs were used at passages 1–4. The cells were cryopreserved and thawed, tested negative for mycoplasma, and exhibited viabilities of 94 ± 3% at the time of the coculture setup.

### 2.3. Flow Cytometric Analysis of the cASC

Flow cytometry was employed for the characterisation of the cASC using antibodies against CD29 (PE, Invitrogen, Carlsbad, CA, USA, 12-0299-42), CD44 (PE, Invitrogen, Carlsbad, CA, USA, 12-0441-82), CD90 (PE, Invitrogen, Carlsbad, CA, USA, 12-5900-42), and CD45 (FITC, Invitrogen, Carlsbad, CA, USA, MA1-80428). An unstained sample was included as the negative control. The data were acquired using a NovoCyte flow cytometer (ACEA Biosciences, San Diego, CA, USA) and analysed using NovoExpress software (version 2.2.0).

### 2.4. U937 and THP-1 Cell Culture and Macrophage Differentiation

The U937 (human histiocytic lymphoma-derived pro-monocytic cell line, Merck KGaA, Darmstadt, Germany, cat. # 85011440-1VL) and THP-1 (human acute monocytic leukaemia cell line, Merck KGaA, Darmstadt, Germany, cat. # 88081201-1VL) cells were cultured in RPMI 1640 medium (Biosera, Cholet, France) supplemented with 10% FBS, 2 mM L-glutamine, 1% penicillin/streptomycin and 1 mM sodium pyruvate (Merck KGaA, Darmstadt, Germany); the THP-1 medium further contained 4.5 g/L D-glucose (Thermo Fisher Scientific, Waltham, MA, USA) and 50 µM β-mercaptoethanol (Merck KGaA, Darmstadt, Germany). The U937 cells were seeded at 5 × 10^5^ cells/mL and the THP-1 cells at 2.5 × 10^5^ cells/mL. For differentiation purposes, both the U937 (1 × 10^6^ cells/well) and the THP-1 (5 × 10^5^ cells/well) cells were treated with 50 ng/mL PMA (Merck KGaA, Darmstadt, Germany) for 96 h and 24 h, respectively, and then cultured in PMA-free medium for 24 h prior to conducting the coculture experiments.

### 2.5. RAW264.7 Cell Culture

The RAW264.7 cells (murine tumour macrophage-like cell line, ATCC, Manassas, VA, USA; cat. # TIB-71) were maintained in DMEM (Biosera, Cholet, France) supplemented with 10% FBS, 2 mM L-glutamine and 1% penicillin/streptomycin. For experimental purposes, 5 × 10^5^ cells were seeded per well in 24-well plates and used the following day.

### 2.6. Generation of Primary Human Macrophages

The peripheral blood monocytes from three donors (male 27, female 42, female 34; cat. # 70025, STEMCELL Technologies Canada Inc., Vancouver, BC, Canada) were cultured in RPMI 1640 medium supplemented as described above for the THP-1 cells and differentiated for 6 days with 50 ng/mL M-CSF (Biomedica Medizinprodukte GmbH, Vienna, Austria; cat. # C-60442). The medium was replaced every 2–3 days.

### 2.7. IL-4/IL-13 Stimulation of the Human Macrophage Cell-Line Models

The U937- and THP-1-derived macrophages were stimulated using IL-4 (H7291, Merck KGaA, Darmstadt, Germany) and IL-13 (SRP3274, Merck KGaA, Darmstadt, Germany) at 25 ng/mL each for 24 and 48 h. Following incubation, the cells were lysed for RNA isolation and qPCR analyses. The experiments were performed in triplicate.

### 2.8. Coculture of the ASCs and Macrophages

The ASCs were seeded on 0.4 μm PET inserts (24-well plate format; cellQART®, SABEU GmbH & Co. KG, Northeim, Germany; cat. no. 9320402) with a surface area of 1.1 cm^2^ to achieve the indicated ratios. The ratios were calculated based on the number of macrophages present at the coculture setup. The macrophages were stimulated with LPS (Lipopolysaccharides from Pseudomonas aeruginosa 10, cat. # L9143, Merck KGaA, Darmstadt, Germany) at 100 ng/mL for the RAW264.7- and U937-derived macrophages, and at 1000 ng/mL for the THP-1-derived macrophages. The LPS concentrations were selected in a model-specific manner to obtain the detectable and reproducible induction of the inflammatory transcripts under the tested culture conditions. An amount of 1000 ng/mL *P. aeruginosa* LPS was selected for the THP-1-derived macrophages since this concentration is within the range used in previously published THP-1/*P. aeruginosa* LPS studies [17]. However, since the U937-derived macrophages and the RAW264.7 cells were stimulated with 100 ng/mL LPS, the magnitude of the ASC-mediated suppression should not be interpreted as being directly comparable between the macrophage models.

The ASC-to-macrophage ratios were: RAW264.7—1:1 (i.e., 5 × 10^5^ ASCs/transwell and 5 × 10^5^ RAW264.7/well), 1:2 (i.e., 2.5 × 10^5^ ASCs/transwell and 5 × 10^5^ RAW264.7/well) and 1:5 (i.e., 1 × 10^5^ ASCs/transwell and 5 × 10^5^ RAW264.7/well); U937—1:2 (i.e., 5 × 10^5^ ASCs/transwell and 1 × 10^6^ U937/well); and THP-1—1:1 (i.e., 5 × 10^5^ ASCs/transwell and 5 × 10^5^ THP-1/well). The ratios were adjusted in order to account for the differences in the cell sizes and plating requirements across the models and to avoid overconfluence or stress of the ASCs on the transwell. The cocultures were performed for 24 and 48 h; these time points were chosen aimed at capturing the early and intermediate transcriptional responses while maintaining feasibility for routine screening purposes. Following incubation, the macrophages were harvested for RNA isolation. Each of the conditions was tested in the form of at least three independent biological replicates, as indicated in the figure legends.

### 2.9. Quantitative PCR (qPCR)

The total RNA was extracted using an RNeasy Mini Kit (QIAGEN GmbH, Hilden, Germany) according to the manufacturer’s instructions. The RNA concentration and purity were assessed using NanoDrop. The cDNA was synthesised from 1 µg of total RNA using a High-Capacity cDNA Reverse Transcription Kit (cat. #4368814, Thermo Fisher Scientific). The quantitative PCR was performed using a TaqMan™ Fast Advanced Master Mix (cat. #4444557, Thermo Fisher Scientific) on a QuantStudio 3 Real-Time PCR System (Thermo Fisher Scientific). Each 20 µL reaction contained cDNA that corresponded to 5 ng reverse-transcribed RNA, the TaqMan Fast Advanced Master Mix, the respective TaqMan Gene Expression Assay and nuclease-free water. The reactions were run in technical duplicates applying the following programme: 95 °C for 20 s, followed by 40 cycles of 95 °C for 1 s and 60 °C for 20 s. No-template and no-reverse-transcription controls were included.

Concerning the human macrophage models, the target mRNAs for the M2 activation comprised *TGFB1* (Hs00998133_m1), *IL10* (Hs00961622_m1), *CCL22* (Hs01574247_m1), *CD209* (Hs01588349_m1) and *IL1RA* (Hs00893626_m1). The inflammatory markers were: *TNFA* (Hs00174128_m1), *Tnfa* (Mm00443258_m1 for RAW264.7), *IL1B* (Hs01555410_m1), *Il1b* (Mm00434228_m1 for RAW264.7) and *PTGS2* (Hs00153133_m1), *Ptgs2* (Mm00478374_m1 for RAW264.7). The expression was normalised to *GAPDH*/*Gapdh* (Hs99999905_m1; Mm99999915_g1 for RAW264.7) and calculated applying the 2^–ΔΔCt^ method. The use of *GAPDH*/Gapdh alone is acknowledged as a limitation, since an assay qualification should include the validation of additional reference genes under inflammatory stimulation.

### 2.10. Statistical Analysis

The statistical analyses were performed in R (version 4.5.3). The statistical testing was performed on the log2-transformed values, whereas the figures show the transformed percentage values for biological interpretability purposes.

The data were analysed separately for each cell line, gene and time point. Once all the treatment conditions were generated within the same independent experimental run, the data were treated as a matched/randomised-block design, with the inclusion of the experimental run as a blocking factor. The log2-transformed values were analysed for the experiments with three or more treatment conditions applying the following model:

log2 expression ~ experimental run + condition

The overall effect of the condition was assessed by means of randomised-block ANOVA. Planned post hoc contrasts were then performed for the biologically relevant conditions, typically untreated macrophages versus stimulated macrophages and stimulated macrophages versus stimulated macrophages cocultured with the ASCs. The contrasts were obtained from the estimated marginal means, and the *p* values were adjusted for multiple comparisons applying the Holm method.

Normality was assessed applying the Shapiro–Wilk test on the model residuals. If the residuals did not meet the normality assumption, the corresponding non-parametric matched analysis was employed, i.e., the Friedman test followed by paired Wilcoxon signed-rank planned comparisons with the Holm adjustment. Paired t-tests were used for the datasets that contained only two matched conditions when the paired differences were compatible with normality; otherwise, Wilcoxon signed-rank tests were applied.

The data were presented as the mean ± SD; the individual biological replicates were shown as single plotted points.

## 3. Results

**Figure 2:**
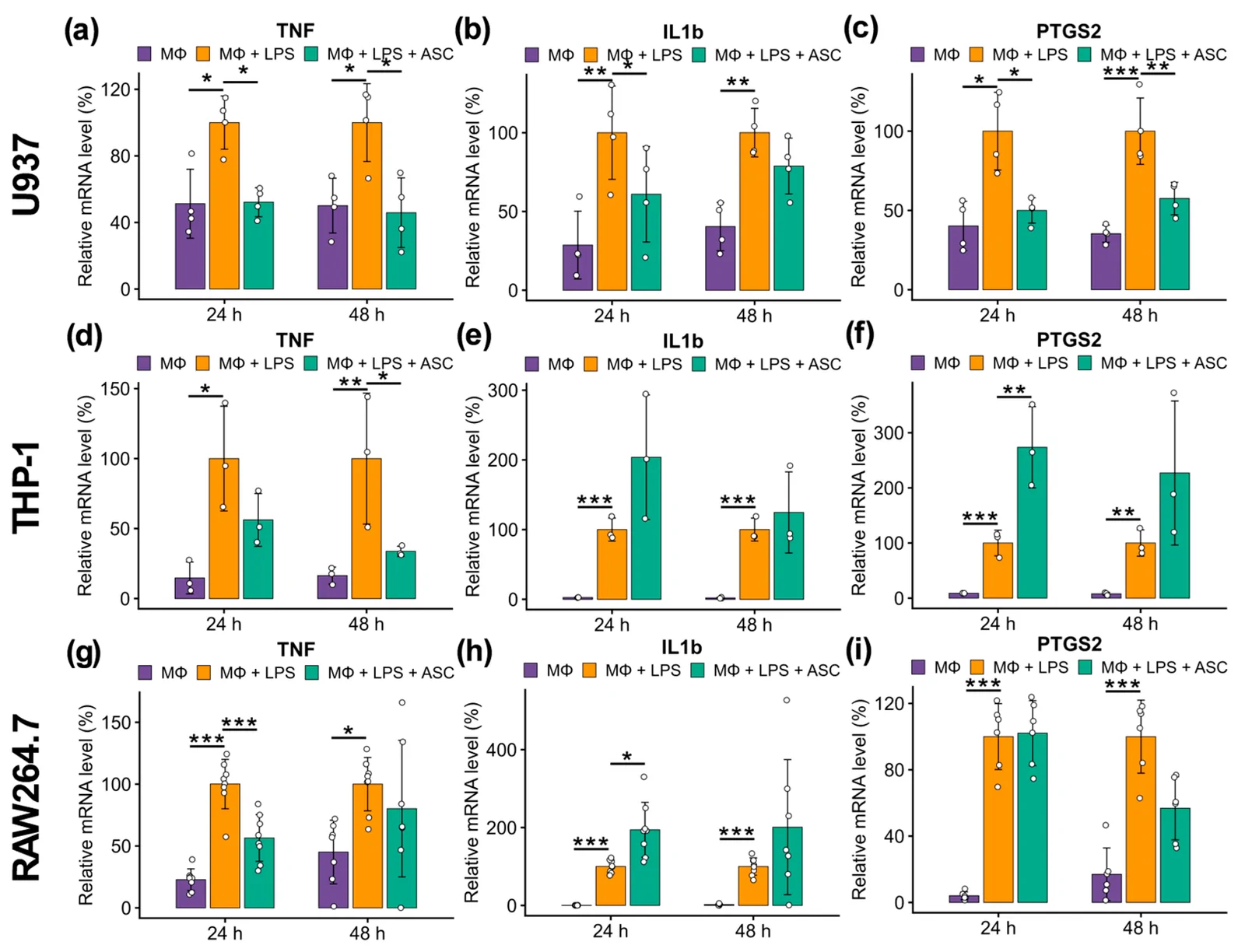
The anti-inflammatory effect of the ASCs seeded in transwell in the various LPS-stimulated macrophage models. (**a**–**c**) The mRNA expression of the inflammatory markers in the U937 macrophages. (**d**–**f**) The mRNA expression of the inflammatory markers in the THP-1 macrophages. (**g**–**i**) The mRNA expression of the inflammatory markers in the RAW264.7 macrophages. (**a**–**f**) The data are presented as the mean relative mRNA expression ± the standard deviation from four independent experiments. (**g**–**i**) The data are presented as the mean relative mRNA expression ± standard deviation from *n* = 7–8 experiments. The data overlap with the data presented in Figure 4 (with the exception of the 1:2 and 1:5 ratios). The statistical analysis was performed applying randomised-block ANOVA, followed by the post hoc testing of the planned contrasts (MΦ versus MΦ + LPS and MΦ + LPS versus MΦ + LPS + ASC). The *p* values were adjusted for multiple testing using the Holm method. The significance is indicated as * *p* ≤ 0.05, ** *p* ≤ 0.01, *** *p* ≤ 0.001; *p* > 0.05 is not displayed.

**Figure 3:**
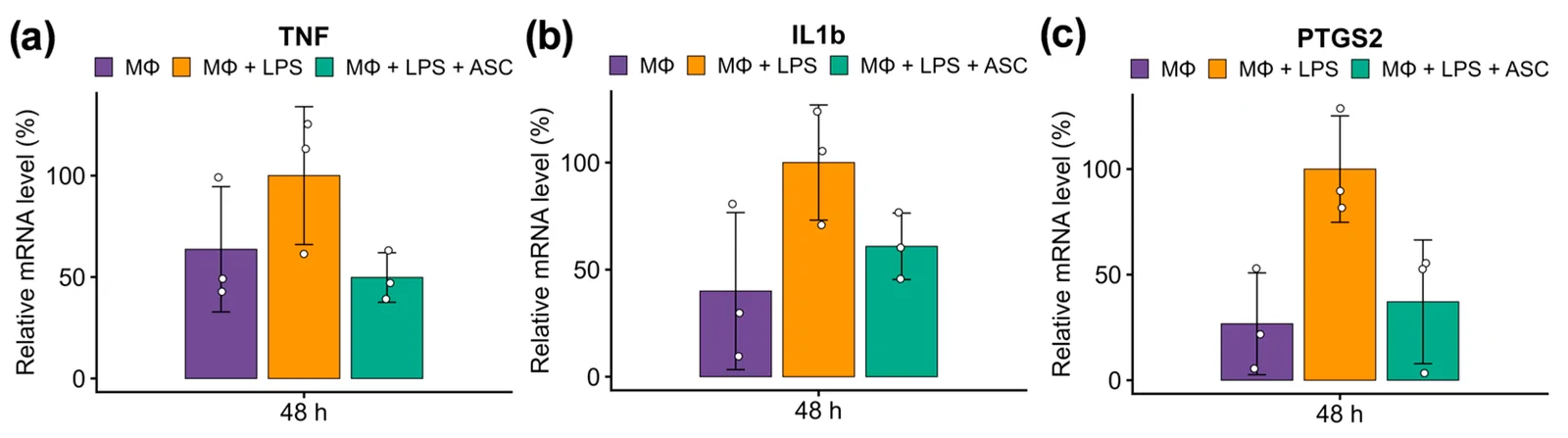
The anti-inflammatory effect of the ASCs cocultured with the human peripheral-blood-monocyte-derived macrophages. The data are presented as the mean relative mRNA expression of (**a**) *TNFA*; (**b**) *IL1B*; (**c**) *PTGS2*; ± the standard deviation from three independent experiments. The statistical analysis was performed applying randomised-block ANOVA, followed by the post hoc testing of the planned contrasts (MΦ versus MΦ + LPS and MΦ + LPS versus MΦ + LPS + ASC). The *p* values were adjusted for multiple testing using the Holm method. Since all the *p* values were >0.05, no statistical significance symbols are displayed.

**Figure A1:**
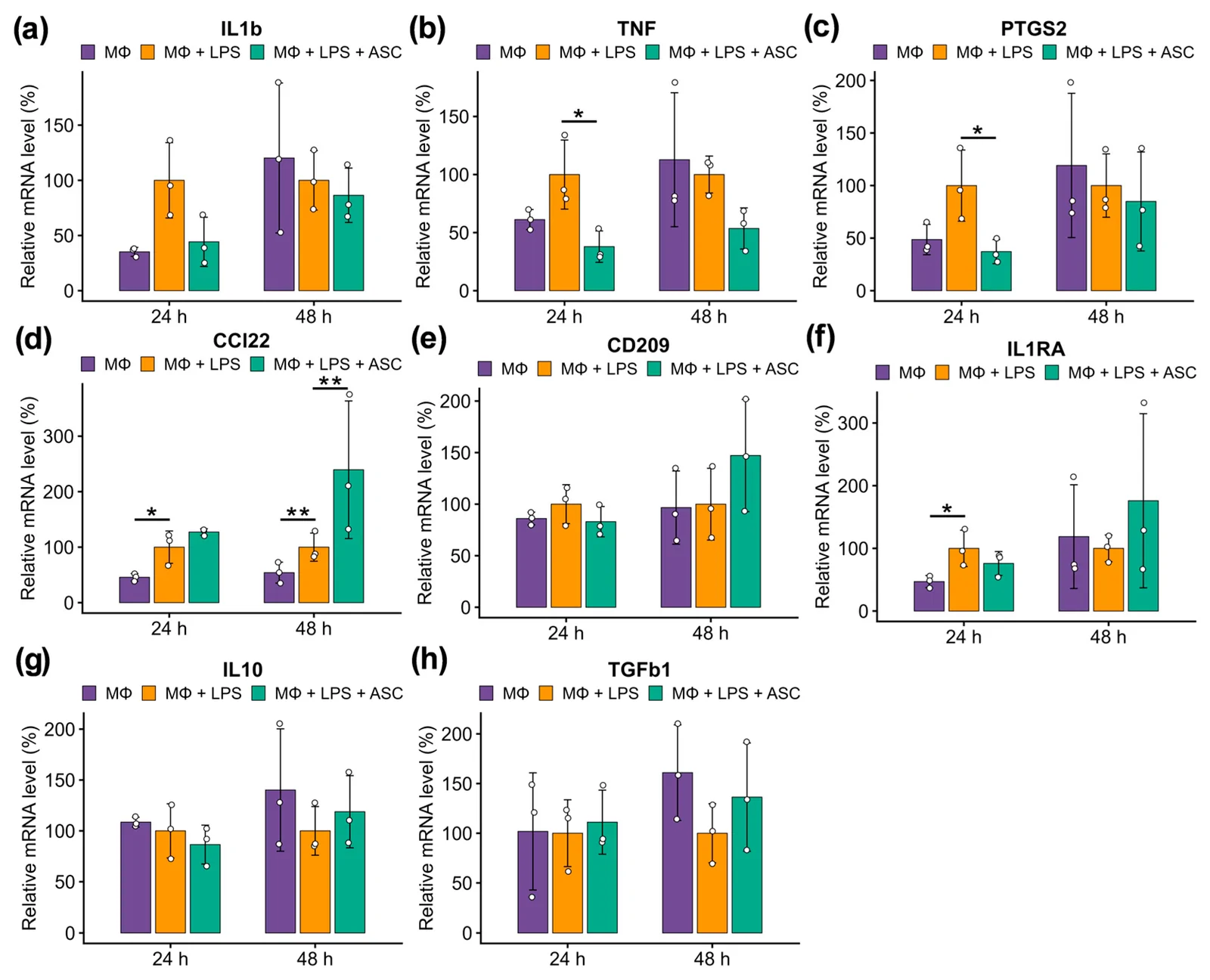
The effect of the ASC cocultivation on the LPS-pre-activated U937 macrophages. The U937 cells were briefly (3 h) pre-activated with LPS prior to seeding the ASC. (**a**–**c**) The effect of the ASC cocultivation on the mRNA expression of the M1 markers. (**d**–**h**) The effect of the ASC paracrine factors on the mRNA expression of the M2 markers. The data are presented as the mean relative mRNA expression ± the standard deviation from three independent experiments. The statistical analysis was performed applying the one-sample t-test (comparison with the reference value of 100%). The statistical analysis was performed applying randomised-block ANOVA, followed by the post hoc testing of the planned contrasts (MΦ versus MΦ + LPS and MΦ + LPS versus MΦ + LPS + ASC). The *p* values were adjusted for multiple testing with the Holm method. The significance is indicated as * *p* ≤ 0.05, ** *p* ≤ 0.01; *p* > 0.05 is not displayed.

The U937-derived macrophages, THP-1-derived macrophages, RAW264.7 macrophages and primary human macrophages were stimulated with LPS in the presence or absence of the ASC coculture using transwell inserts. The mRNA expression of the pro-inflammatory markers was analysed after 24 and 48 h (Figure 2). Although the ASC coculture reduced the selected LPS-induced inflammatory transcripts, the response was not uniform across the macrophage models or markers. *TNFA*, *IL1B* (*p* < 0.05 after 24 h, *p* > 0.05 after 48 h) and *PTGS2* were reduced in the U937-derived macrophages. *TNFA* was reduced in THP-1-derived macrophages, whereas *IL1B* and *PTGS2* were not suppressed and evinced increased transcript levels under the selected conditions. The response in the RAW264.7 cells was again marker-dependent, with a decrease only in *Tnfa*, whereas the *Il1b* expression increased significantly at 24 h relative to the LPS-only condition. The ASC-coculture-derived soluble factors suppressed the pro-inflammatory mRNA expression of *TNFA* and *PTGS2* in the U937 macrophages when applied after a short period (3 h) of LPS pre-activation, thus indicating activity in the already-activated macrophages (Figure A1a–c). A statistically nonsignificant decrease in the expression of the inflammatory markers was detected in the primary human macrophages derived from the peripheral blood monocytes (Figure 3).

**Figure 4:**
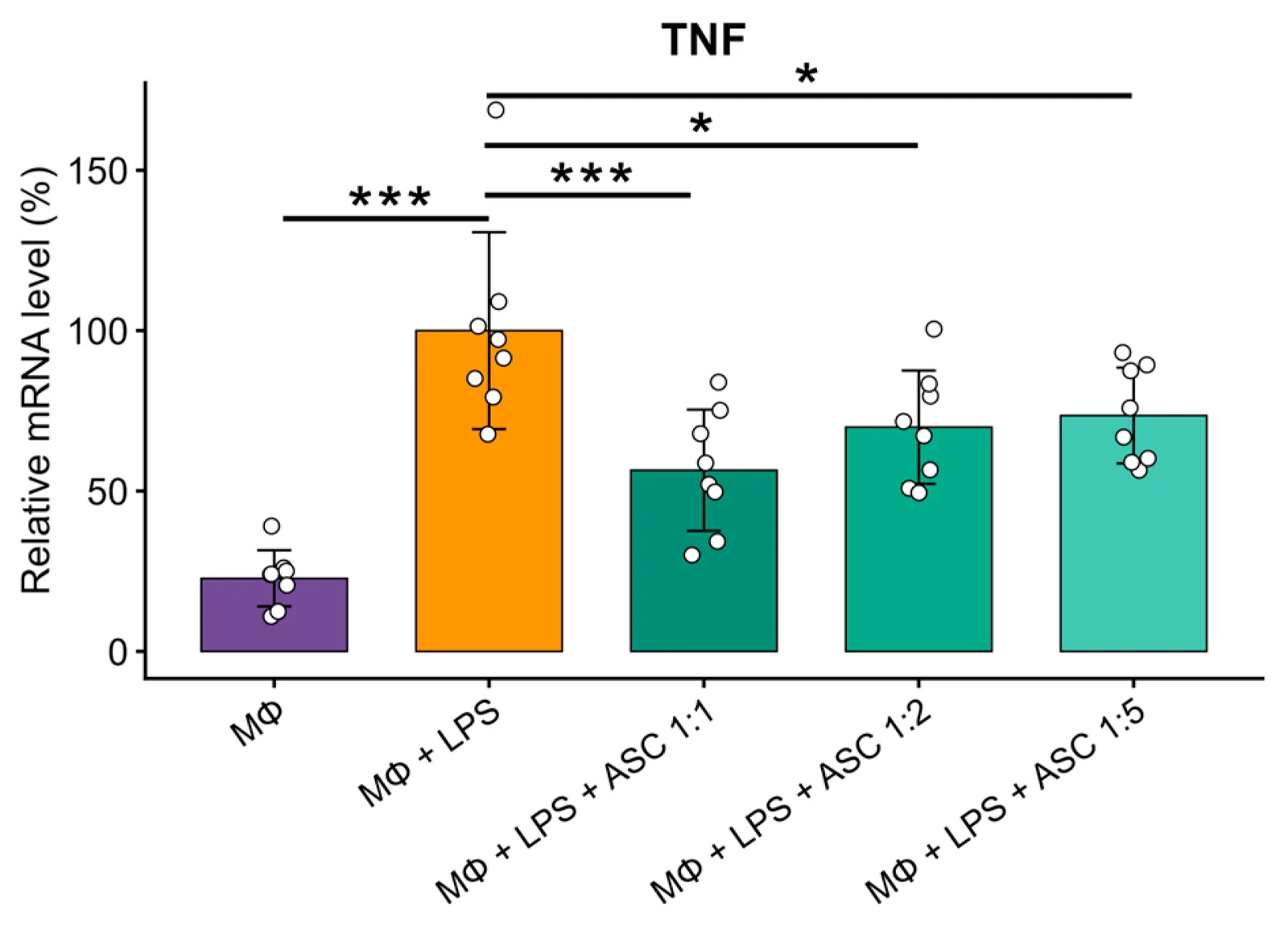
The effect of the AST-to-RAW264.7 ratio on TNFα expression. The RAW264.7 cells were seeded at 5 × 10^5^ cells per well and cocultured with ASCs at ratios of 1:1 (5 × 10^5^ ASCs), 1:2 (2.5 × 10^5^ ASCs), and 1:5 (1 × 10^5^ ASCs). The data are presented as the mean relative mRNA expression ± the standard deviation from eight independent experiments. The statistical analysis was performed applying randomised-block ANOVA, followed by the post hoc testing of the planned contrasts (MΦ versus MΦ + LPS and MΦ + LPS versus MΦ + LPS + ASC in the specified ratios). The *p* values were adjusted for multiple-testing applying the Holm method. The significance is indicated as * *p* ≤ 0.05, *** *p* ≤ 0.001; *p* > 0.05 is not displayed.

Given their adherent growth and lack of a differentiation requirement, RAW264.7 cells were selected to assess the effect of reducing the ASC abundance. The RAW264.7 cells were cocultured at ASC-to-RAW264.7 ratios of 1:1, 1:2, and 1:5; the macrophage number was kept constant. The TNFα expression was lower than in the LPS-only condition at each tested ratio, with the greatest numerical suppression at the 1:1 ratio (Figure 4).

**Figure 5:**
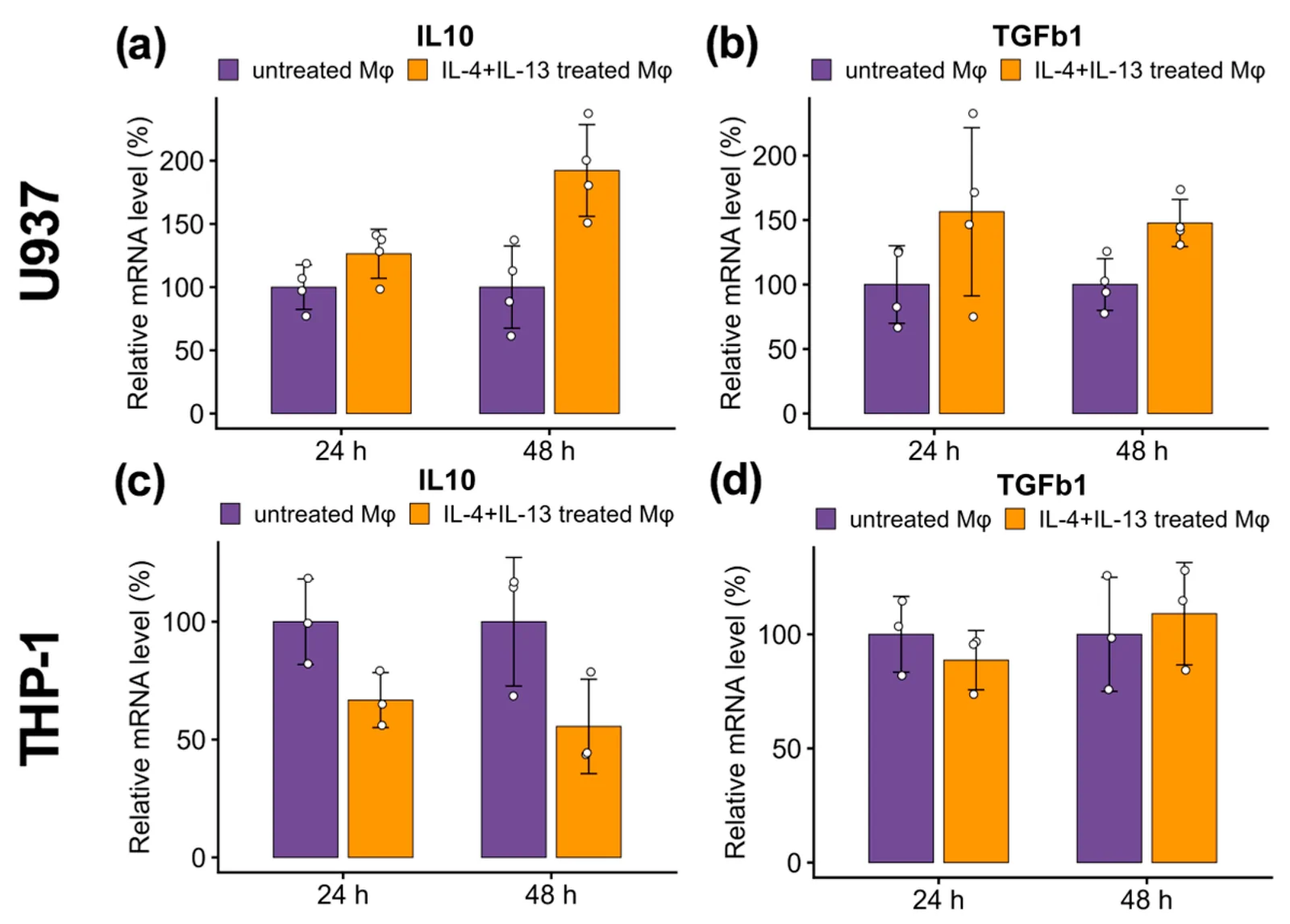
The activation of the THP-1 and U937 macrophage cell lines in the M2 phenotype. (**a**,**b**) *IL10* and *TGFB1* gene expression in the M2 phenotype-stimulated U937 cells and (**c**,**d**) THP-1 cells. The data are presented as the mean relative mRNA expression ± the standard deviation from four (**a**,**b**) or three (**c**,**d**) independent experiments. The values were normalised to the untreated macrophages to show the cytokine-induced polarisation. The statistical analysis was performed using the paired t-test (comparison with the reference groups of the untreated macrophages). Since all the *p* values were >0.05, no statistical significance symbols are displayed.

**Figure 6:**
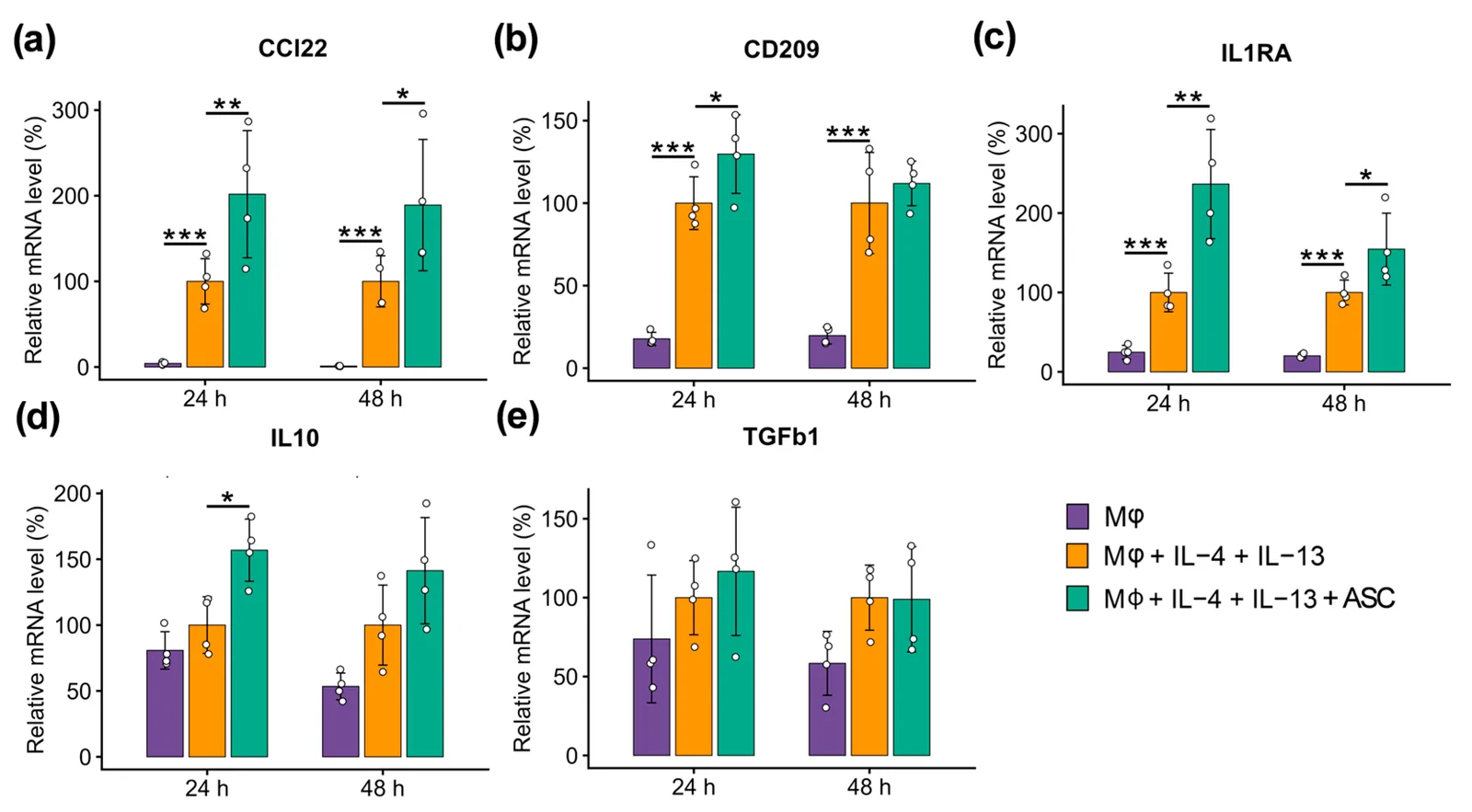
The effect of the ASC coculture on the M2 polarisation of U937. (**a**–**e**) The mRNA expression of the various M2 markers. The data are presented as the mean relative mRNA expression ± the standard deviation from four independent experiments. The values were normalised to the IL-4/IL-13-treated U937 macrophages to show the additional effect of the ASC coculture on the polarised condition. The statistical analysis was performed applying randomised-block ANOVA, followed by the post hoc testing of the planned contrasts (MΦ versus MΦ + IL-4/IL-13 and MΦ + IL-4/IL-13 versus MΦ + IL-4/IL-13 + ASC). The *p* values were adjusted for multiple testing with the Holm method. The significance is indicated as * *p* ≤ 0.05, ** *p* ≤ 0.01, *** *p* ≤ 0.001; *p* > 0.05 is not displayed.

**Figure A2:**
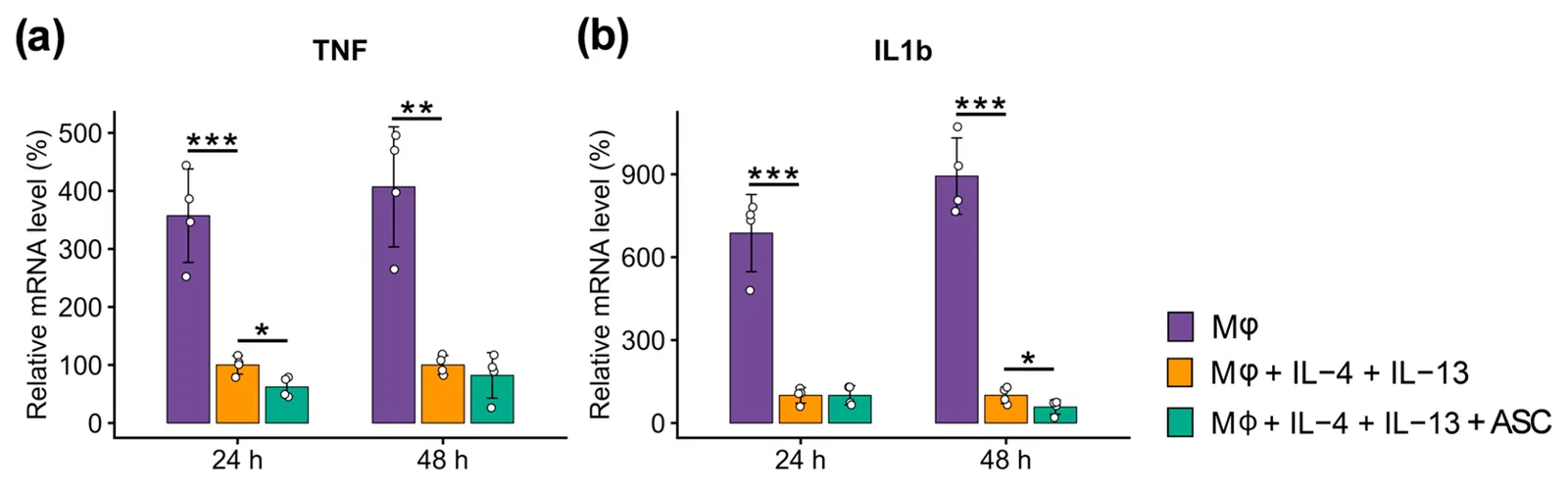
The effect of the ASC cocultivation on the mRNA expression of the M1 markers in the U937 M2 macrophage model. The data are presented as the mean relative mRNA expression ± the standard deviation from four independent experiments. The statistical analysis was performed applying randomised-block ANOVA, followed by the post hoc testing of the planned contrasts (MΦ versus MΦ + IL-4+IL-13 and MΦ + LPS versus MΦ + IL-4+IL-13 + ASC). The *p* values were adjusted for multiple testing with the Holm method. The significance is indicated as * *p* ≤ 0.05, ** *p* ≤ 0.01, *** *p* ≤ 0.001; *p* > 0.05 is not displayed.

Aimed at evaluating whether the human-derived macrophage models were suitable for the assessment of regenerative polarisation, the U937 and THP-1 cells were stimulated with IL-4 and IL-13, which was followed by the analysis of the M2-associated gene expression. Only the U937-derived macrophages exhibited the moderate and statistically non-significant upregulation of *IL10* and *TGFB1* following IL-4/IL-13 stimulation (Figure 5). In contrast, the THP-1 macrophages did not exhibit the reproducible transcriptional induction of these markers under the test conditions. Further analysis of the U937 macrophages demonstrated that the ASC-derived paracrine factors modestly potentiated the IL-4/IL-13-induced M2-associated mRNA expression, including *IL10*, *IL1RA*, *CD209* and *CCL22* (Figure 6). In addition, the ASC coculture further reduced the *TNFA* expression at 24 h and the *IL1B* expression at 48 h; the remaining ASC-coculture comparisons did not reach statistical significance (Figure A2). However, the ASC-derived paracrine factors alone were in most cases insufficient in terms of inducing the M2-associated mRNA expression in the short-term LPS-preactivated U937 macrophages, with the exception of *CCL22* at 48 h (Figure A1d–h).

## 4. Discussion

Although mesenchymal stromal cell (MSC)-based products have been subjected to detailed investigation in the field of regenerative medicine in recent years, their clinical translation remains limited due to suboptimal dosing and timing and a lack of standardised manufacturing and quality control approaches [2,4,18,19,20]. While MSCs exhibit well-established immunomodulatory and anti-inflammatory effects, their successful clinical implementation depends on the optimisation of robust potency assays, the dosing strategy and consistent product characterisation [21,22].

Potency testing has become a key component in terms of the evaluation of MSC products. Traditional T-cell proliferation assays are beset with challenges surrounding both their variability and limited reproducibility [9]. Recent evidence indicates that macrophages (rather than lymphocytes) are the primary immune targets of MSC products and their secretomes [13]. Consequently, macrophage-based assays are gaining relevance, especially with regard to conditions such as wound healing and osteoarthritis, in which macrophages orchestrate both the inflammatory and reparative processes [11,23,24].

THP-1, U937 and RAW264.7 rank amongst the most commonly applied macrophage models. THP-1 and U937 are human monocyte-derived cell lines with distinct differentiation kinetics, while RAW264.7 cells constitute an immortalised murine macrophage line with a relatively pro-inflammatory, M1-like baseline phenotype. M2 polarisation, which is typically induced by IL-4 and IL-13, is used to assess the pro-regenerative potential; however, the degree of efficiency of polarisation varies substantially across cell lines [25,26,27,28]. In line with this observation, the polarisation responses differed markedly across the tested models, as previously reported in studies that determined substantial heterogeneity between commonly used macrophage lines. THP-1 and U937 differ in terms of their differentiation protocols and basal activation states, which potentially significantly condition cytokine-driven transcriptional programmes. *IL10* and *TGFB1* were selected as the initial screening markers for the comparison of the responsiveness of IL-4 and IL-13 across the two human macrophage cell-line models. Since the THP-1 cells did not evince the reproducible induction of these first-line markers under the tested conditions, the extended M2-associated panel was applied only to the U937-derived macrophages. Although the U937-derived macrophages evinced upregulation in the expression of *IL10* and *TGFB1*, these changes were not statistically significant. Therefore, the conclusion that the U937 cells constituted the most suitable model for the M2-associated transcriptional readouts was limited to this observation and the stepwise screening strategy. Together, these observations reinforce the fact that macrophage models are not functionally interchangeable and should be benchmarked and validated prior to their use for standardised potency testing purposes.

Although this study confirmed the anti-inflammatory role of the ASC coculture in various macrophage models, it also highlighted certain line-specific responses. The *IL1B* expression was significantly reduced in the U937-derived macrophages at 24 h, and the suppressive trend was maintained at 48 h, although the latter comparison did not reach statistical significance, which possibly reflected differences in the LPS sensitivity or higher intrinsic pro-inflammatory bias in the other lines. Similarly, the *PTGS2* expression in the THP-1 cells was observed not to be suppressed, most likely due to stronger LPS-induced activation. The primary human macrophages evinced a similar trend in the reduction in the selected pro-inflammatory transcripts; however, the response was not statistically significant.

Since the macrophage models differed in terms of cell size, adherence, differentiation requirements and culture density tolerance, model-specific ASC-to-macrophage ratios were used to avoid ASC overconfluence or macrophage stress. Consequently, the magnitude of the ASC-mediated suppression should not be interpreted as a direct quantitative comparison between the macrophage models. Rather, the data identified candidate responder models and readouts under feasible culture conditions. Future assay qualification should include a harmonised reference ratio or an experimentally justified ratio-response matrix.

The higher LPS concentration used for the THP-1-derived macrophages represents a significant limitation of the study. Although 1000 ng/mL *P. aeruginosa* LPS is within the published range for THP-1-based macrophage activation [17], the use of a ten-fold higher stimulus than in the U937 and RAW264.7 cells prevented the direct comparison of the anti-inflammatory effect size across the models. The stronger inflammatory stimulus may have contributed to the THP-1-specific pattern in which *TNFA* was reduced, whereas the *IL1B* and *PTGS2* were not suppressed and instead exhibited increased transcript levels following the ASC coculture. A directionally opposite response was also detected in the RAW264.7 cells, in which ASC coculture significantly increased the *Il1b* expression at 24 h. This result indicates that the paracrine effect of the ASCs cannot be characterised as uniformly anti-inflammatory across markers or responder models. The increase may reflect model-specific signalling under the selected *P. aeruginosa* LPS conditions, but its biological basis cannot be established from transcriptional data alone and requires protein-level and mechanistic follow-up research.

The use of *P. aeruginosa*-derived LPS should be considered when interpreting the data. The LPS structure and source potentially bias TLR4 signalling and macrophage transcriptional responses [29]; therefore, the observed marker patterns are not necessarily identical to those obtained with E. coli-derived LPS. Future assay qualification should include a defined and standardised inflammatory stimulus and, where relevant, the comparison of the LPS sources.

Only the U937 macrophages responded to IL-4 and IL-13 stimulation, with the upregulation of *IL10* and *TGFB1*. However, the IL-4/IL-13 response in the U937-derived macrophages was moderate, statistically non-significant and only at the transcriptional level. A higher increase in the M2-associated markers was detected in the U937 macrophages following cocultivation with the ASCs, which provided support for the use of this experimental setup. Thus, the term “M2 polarisation” should be interpreted cautiously; the data support IL-4/IL-13-responsive M2-associated marker induction rather than a fully validated M2 phenotype.

The main aim of this study was to compare various cell lines that are suitable for the routine biological testing of MSC-based products. It was not our intention to demonstrate pronounced phenotypic shifts, but rather to evaluate the sensitivity and consistency of various macrophage models as readouts for the activity of the MSC coculture. In this context, the modest yet reproducible transcriptional changes in the key immunoregulatory markers are informative in terms of the assessment of comparative potency as opposed to a mechanistic interpretation.

The study did not include a non-MSC stromal-cell control, such as fibroblasts. Therefore, the observed effects should be interpreted as ASC-associated paracrine effects under the tested conditions and not as proof that these effects are unique to ASCs as compared to other adherent stromal cells.

The exclusive reliance on transcriptional readouts is a further limitation of the study. Although qPCR-based assays provide a high degree of sensitivity and are well-suited to scalable screening workflows, mRNA levels do not necessarily reflect the protein expression or biological function. Therefore, the considered assay should be regarded as a preliminary transcriptional screening tool. Additional validation at the protein level (e.g., the quantification of secreted cytokines such as TNFα, IL-1RA and IL-10, surface markers such as CD163/CD206/CD209 where species-appropriate antibodies are available) and functional assays such as phagocytosis or the suppression of inflammatory mediator release are required in order to be able to fully characterise the polarisation states. Moreover, intervention-based approaches such as neutralisation or blocking experiments are required to identify the specific mediators within the ASC secreted paracrine factors that are responsible for the observed effects. Since our study was designed to provide a comparative evaluation of selected macrophage models rather than a mechanistic investigation, these aspects were beyond the scope of the research and should be addressed in future studies.

From the translational perspective, the proposed macrophage-based platform represents progress towards the development of a functionally oriented potency assay, which requires species-matched systems, orthogonal protein or functional readouts, and a formal analytical validation prior to its use in batch release. The incorporation of a biologically meaningful readout that reflects the immunomodulatory activity of the MSC paracrine effects would complement the established identity, purity and viability criteria and provide support for a more comprehensive evaluation of product consistency. Such an approach would be particularly valuable given the well-documented donor-to-donor and manufacturing-associated variability of MSC preparations.

The xenogeneic nature of the considered assay is an important limitation. The cross-species approach was intentionally adopted as a pragmatic, exploratory strategy for the evaluation of the robustness of assays across distinct responder systems. However, direct canine–human macrophage comparisons indicate that macrophage activation is partly conserved across species but not interchangeable [30]. Canine macrophages have been reported to evince greater sensitivity to IFN-γ activation than human macrophages, as reflected by the co-stimulatory molecule expression and TNFα production [31]. According to side-by-side M1/M2a activation studies, canine and human macrophages exhibited similar induction of selected M1-associated genes, whereas the M2-associated transcripts, including *IL10*, *CCL22*, *TGFB* and *CD163*, displayed more species-dependent patterns. Therefore, canine ASC-derived paracrine mediators potentially do not engage human or murine macrophage receptors, downstream signalling pathways, or polarisation programmes with the same efficacy as in a species-matched canine system. This was particularly relevant with concern to the LPS-preactivated U937 experiment, in which the absence of M2-associated marker induction should be interpreted as a limitation of the xenogeneic, inflammatory, short-term transcriptional setup rather than as evidence that canine ASCs lack M2-modulating activity. The future development of assays for veterinary translation should include species-matched canine macrophage models, such as primary canine monocyte-derived macrophages or validated canine macrophage cell lines.

Although ASCs from multiple canine donors were used, the study was not designed or powered to quantify the donor-to-donor variability in ASC paracrine potency. Donor-related variability remains an important factor for future assay qualification.

## 5. Conclusions

This study compared macrophage models as transcriptional readouts for the anti-inflammatory paracrine activity of canine ASCs. The U937-derived macrophages evinced the most consistent suppression of LPS-induced inflammatory transcripts and represented the only model that exhibited moderate but non-significant IL-4 and IL-13-responsive M2-associated transcriptional changes under the test conditions. The RAW264.7 cells provided practical advantages for routine culture and selected anti-inflammatory screening readouts, whereas the THP-1 cells exhibited important marker-specific limitations, including a lack of IL1β and PTGS2 suppression under the selected high-LPS condition. Together, these findings support the use of macrophage-based transcriptional assays for early-stage model benchmarking and candidate readout selection purposes. However, the considered xenogeneic, qPCR-based assay does not represent a validated potency-release test. Future development will require species-matched validation, protein-level and functional readouts, donor-variability assessments, and formal analytical qualification before use in the testing of veterinary or human cell-therapy products.

## Data Availability

The data generated in the study are available at https://doi.org/10.6084/m9.figshare.33245751.

## Abbreviations

The following abbreviations are used in this manuscript:

ASCs	Adipose-derived mesenchymal stromal cells
GvHD	Graft-versus-host disease
LPS	Lipopolysaccharide
MLR	Mixed lymphocyte reaction
MSCs	Mesenchymal stromal cells
MΦ	Macrophage
PBMC	Peripheral blood mononuclear cells
PMA	Phorbol 12-myristate 13-acetate

## Appendix A

**Table A1:** Details of the ASC donors and the flow cytometry characterisation of the ASCs. The flow cytometry of the surface CD markers indicates relative positivity [%].

Breed	Sex	Age	Weight	CD29	CD44	CD45	CD90
Yorkshire Terrier	Female	2 years	3.5 kg	99.88	99.89	6.21	98.58
Beagle	Female	1 year	15 kg	98.83	95.66	1.76	98.49
Border Collie	Female	1 year	11.7 kg	99.87	98.67	0.54	99.75
